# Modification of Hydrophilic and Hydrophobic Surfaces Using an Ionic-Complementary Peptide

**DOI:** 10.1371/journal.pone.0001325

**Published:** 2007-12-19

**Authors:** Hong Yang, Shan-Yu Fung, Mark Pritzker, P. Chen

**Affiliations:** Department of Chemical Engineering, University of Waterloo, Waterloo, Ontario, Canada; United States Naval Research Laboratory, United States of America

## Abstract

Ionic-complementary peptides are novel nano-biomaterials with a variety of biomedical applications including potential biosurface engineering. This study presents evidence that a model ionic-complementary peptide EAK16-II is capable of assembling/coating on hydrophilic mica as well as hydrophobic highly ordered pyrolytic graphite (HOPG) surfaces with different nano-patterns. EAK16-II forms randomly oriented nanofibers or nanofiber networks on mica, while ordered nanofibers parallel or oriented 60° or 120° to each other on HOPG, reflecting the crystallographic symmetry of graphite (0001). The density of coated nanofibers on both surfaces can be controlled by adjusting the peptide concentration and the contact time of the peptide solution with the surface. The coated EAK16-II nanofibers alter the wettability of the two surfaces differently: the water contact angle of bare mica surface is measured to be <10°, while it increases to 20.3±2.9° upon 2 h modification of the surface using a 29 µM EAK16-II solution. In contrast, the water contact angle decreases significantly from 71.2±11.1° to 39.4±4.3° after the HOPG surface is coated with a 29 µM peptide solution for 2 h. The stability of the EAK16-II nanofibers on both surfaces is further evaluated by immersing the surface into acidic and basic solutions and analyzing the changes in the nanofiber surface coverage. The EAK16-II nanofibers on mica remain stable in acidic solution but not in alkaline solution, while they are stable on the HOPG surface regardless of the solution pH. This work demonstrates the possibility of using self-assembling peptides for surface modification applications.

## Introduction

Surface modification plays an important role in materials science and interfacial engineering and has been widely used to improve the compatibility of given materials with other materials or environments. For example, biocompatibility is an important issue for materials used for transplantation and bioanalytical applications [1], [2]. For such a purpose, the material surface usually has to be physically/chemically modified to improve its compatibility with the human body [3], [4]. Surface modification can also impart additional functionalities to the materials; in particular, smart drug delivery vehicles have benefited from surface modification for cellular targeting and membrane penetration [5]–[7].

Recent advances in surface modification have had a significant impact on the development of microfluidic bioanalysis [8], immobilization of enzymes and DNA for the purpose of developing biocompatible biosensors [9] and the control of cell adhesion in tissue engineering and biomedical applications [10], [11]. One achievement is the conjugation of both polyethylene glycol (PEG) and tri-peptide Arg-Gly-Asp (RGD) on polymethyl methacrylate (PMMA) to control cell adhesion and growth for keratoprosthesis applications [12]. Another is the surface modification of a conductive gold surface with a protein fragment containing a cell adhesion domain in order to grow and pattern neurons for potential use in multi-electrode array recording technology [13]. Furthermore, the use of a dipeptide Phe-Phe (FF) to modify graphite and gold electrodes has improved the electrochemical reactivity of these surfaces to detect hydrogen peroxide and NADH [14], [15].

Among the many emerging materials, self-assembling peptides show promise for use in surface modification. In this category is a special class of ionic-complementary peptides that contain sequences derived from a fragment of a Z-DNA binding protein in yeast [16]. They have unique sequences with alternating hydrophobic and hydrophilic residues, imparting them with amphiphilic characteristics. They can self-assemble into β-sheet-rich nanofibrils and macroscopic membranes. These β-sheets were found to be very stable over a wide range of pHs (1.5–11), at high temperature (up to 90°C) and in the presence of denaturation agents (e.g., 0.1% SDS and 8 M urea) and proteases [16], [17]. These peptides have been studied for a wide number of applications, ranging from tissue scaffolding, biological surface patterning to drug delivery [16], [18]–[25]. A special design of these peptides can form a self-assembled monolayer (SAM) on gold surfaces and facilitate cell patterning for purposes of investigating cell communications and signal transduction [26].

The ionic-complementary peptides have several features that are advantageous for modifying surfaces. First, their amphiphilic nature allows them to interact with both hydrophobic and hydrophilic surfaces. Second, simple adjustment of the solution pH can alter the charge state of the peptide residues and consequently their interaction with the surface and peptide assembly on the surface. Third, the peptide sequence can be designed and synthesized to incorporate certain amino acids with desired functions, such as RGD for cell attachment [12] and residues containing COOH [27] and NH_2_ [28] groups for protein immobilization. Fourth, the self-assembled peptide nanostructures exhibit excellent chemical stability [17] with good *in vitro* and *in vivo* biocompatibility [16], [21], [24], [29]. With these promising features, self-assembling peptide modified surfaces have great potential for use in (bio)molecular sensing and tissue engineering.

**Figure 1:**
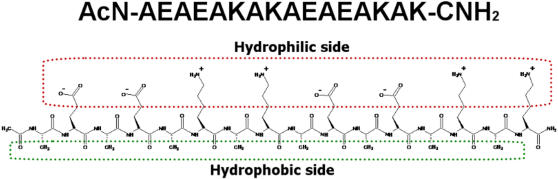
Schematic diagram of EAK16-II structure. The red portion shows the hydrophilic side of the peptide and the green portion shows the hydrophobic side of the peptide.

In this study, we demonstrate how a model ionic-complementary peptide EAK16-II (Figure 1) assembles on a hydrophilic (mica) and a hydrophobic (HOPG) surface. This peptide has been extensively studied in our group [30]–[32]. We take this research one step further by examining how the assembly of EAK16-II modifies mica and HOPG surfaces. The operation of AFM in liquid is the primary method used to observe the peptide assembly on the model surfaces. The hydrophobicity of the peptide-modified surfaces is characterized by water contact angle measurements. The stability of the modified surfaces in acidic and alkaline solutions is evaluated in terms of the surface coverage changes determined from AFM image analysis. The information obtained in this study is aimed at providing a more complete picture of the peptide assembly on both hydrophilic and hydrophobic surfaces and investigating whether peptides such as EAK16-II can be used to modify electrodes to enhance the biocompatibility of the surface for the immobilization of biomolecules and further use as (bio)molecular sensors.

## Results and Discussion

### EAK16-II assembly on hydrophilic mica surface

**Figure 2:**
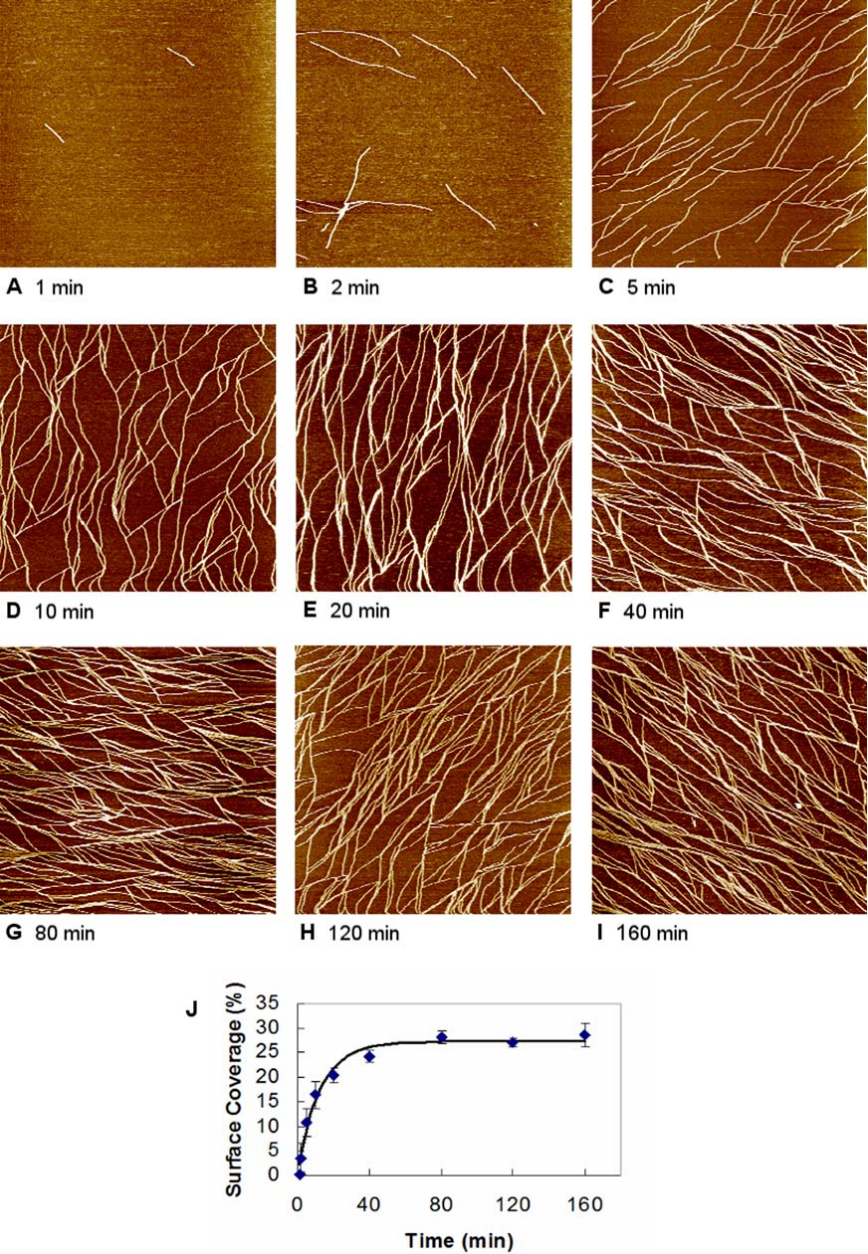
Time evolution of 4 µM EAK16-II assembly on the mica surface. AFM images collected at various incubation times: (a) 1 min; (b) 2 min; (c) 5 min; (d) 10 min; (e) 20 min; (f) 40 min; (g) 80 min; (h) 120 min; (i) 160 min. Surface coverage of EAK16-II on mica as a function of incubation time is plotted in panel (j). Each image corresponds to a scan area of 2000 nm×2000 nm.

The AFM images (Figure 2 panels a–i) show that EAK16-II can adhere to a mica surface and assemble into nanofibers after a certain time in pure water. In the presence of 4 µM EAK16-II, a few single and unbranched short fibers are observed after 1 min of incubation (Figure 2a). With time, the fibers appearing on the mica surface become longer and more numerous (Figures 2b and c). After 10 min, fiber networks are observed (Figures 2d–i). The evolution of the surface coverage of EAK16-II nanofibers with the incubation time is plotted in Figure 2j. The surface coverage of EAK16-II nanofibers increases rapidly with time over the first 30 or 40 min before leveling off to a plateau of about 27% after ∼1 h, indicating that saturation has been reached.

**Figure 3:**
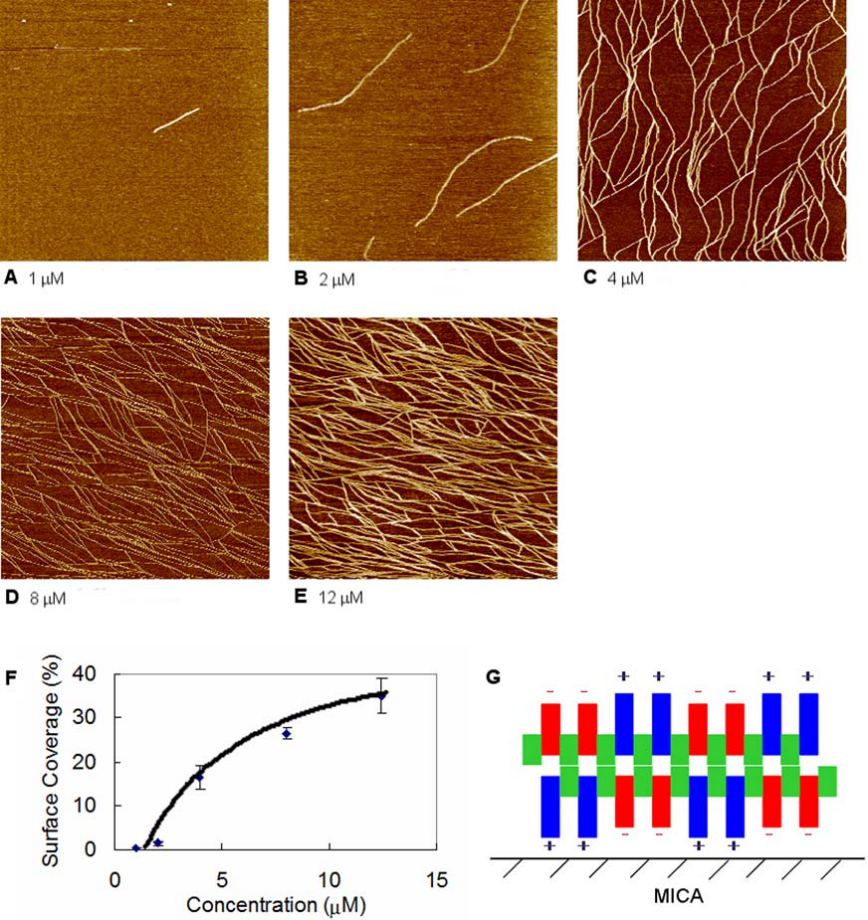
Effect of EAK16-II concentration on the surface coverage of peptide assemblies on mica. AFM images of EAK16-II-modified mica surfaces at 10 min incubation in solutions with various peptide concentrations: (a) 1 µM; (b) 2 µM; (c) 4 µM; (d) 8 µM; (e) 12 µM. Surface coverage of EAK16-II nanofibers on mica as a function of peptide concentration is plotted in panel (f). Proposed molecular arrangement of EAK16-II assembly on mica is plotted in panel (g). Each image corresponds to a scan area of 2000 nm×2000 nm.

As expected, the surface coverage of EAK16-II nanofibers on mica is affected by the peptide concentration. Figure 3 shows images obtained at different peptide concentrations after an incubation time of 10 min. Only a few short fibers are observed on mica at a low EAK16-II concentration of 1 µM (Figure 3a). When the EAK16-II concentration is raised to 2 µM, the number and length of the nanofibers increase (Figure 3b). A further rise in the concentration to above 4 µM leads to the formation of fiber networks, which become denser as the peptide concentration continues to increase (Figure 3c–e). Quantitative analysis of the images yields the variation in surface coverage of EAK16-II nanofibers with peptide concentration shown in Figure 3f. This indicates that the peptide concentration can be used to control its surface coverage on mica.

The width and height of the nanofibers are observed to be independent of peptide concentration and incubation time, and estimated to be ∼7.0±2.6 nm and ∼1.8±0.2 nm, respectively, after AFM tip deconvolution [30]. These dimensions are likely affected by the molecular arrangement of EAK16-II in the nanofibers and so form the basis for the proposed structure shown in Figure 3g. It has been reported that EAK16-II forms β-sheets in pure water and salt solutions [16], [17], [32]. In a β-sheet structure, successive side chains of a polypeptide extend to opposite sides of the pleated sheet, resulting in a distance of ∼0.7 nm between two adjacent residues on one side of the sheet [33]. A 16-residue β-strand would therefore have a length of 5.6 nm. Considering the N and C-terminal protection groups contribute an additional length of about 0.4 nm (estimated from Chemsketch software, Toronto, Canada), the total length of an EAK16-II β-strand would be approximately 6 nm. This length is close to the fiber width (7.0±2.6 nm) observed on the mica surface. The height of a single EAK16-II molecule in a β-strand conformation is ∼0.9 nm (estimated from Chemsketch software). By comparing the height of a single EAK16-II molecule with that of the EAK16-II nanofibers, we may infer that the nanofibers on mica surface are mostly made of two β-sheet layers stacked on top of one another. With such a structure, charged residues of the bottom layer peptide are oriented toward the hydrophilic mica surface and those of the top layer peptide toward the aqueous solution. The two β-sheet layers are held together by interactions between the hydrophobic residues of the two EAK16-II molecules. This arrangement minimizes the contact of the hydrophobic residues with either mica or the solution.

In the proposed model (Figure 3g), the modification of the mica surface by the peptide nanofibers is due to EAK16-II affinity for the surface. The negatively charged mica surface can attract positively charged lysine residues at neutral pH. This electrostatic attraction is expected to be the major driving force for the adsorption and adhesion of the peptide on mica to be discussed in a later section. Since a chemical bond between the peptide and the mica surface is unlikely to form and the peptide nanofibers remain on mica after rinsing three times, the adhesion of the peptide to mica surface may be classified as physical adsorption. The growth of short peptide nanofibers into long fibers and fiber networks is thought to occur by the attachment of monomers and small protofibers onto the fiber ends at the mica surface, which has been characterized as surface-assisted nanofiber growth [34].

### EAK16-II assembly on hydrophobic HOPG surface

**Figure 4:**
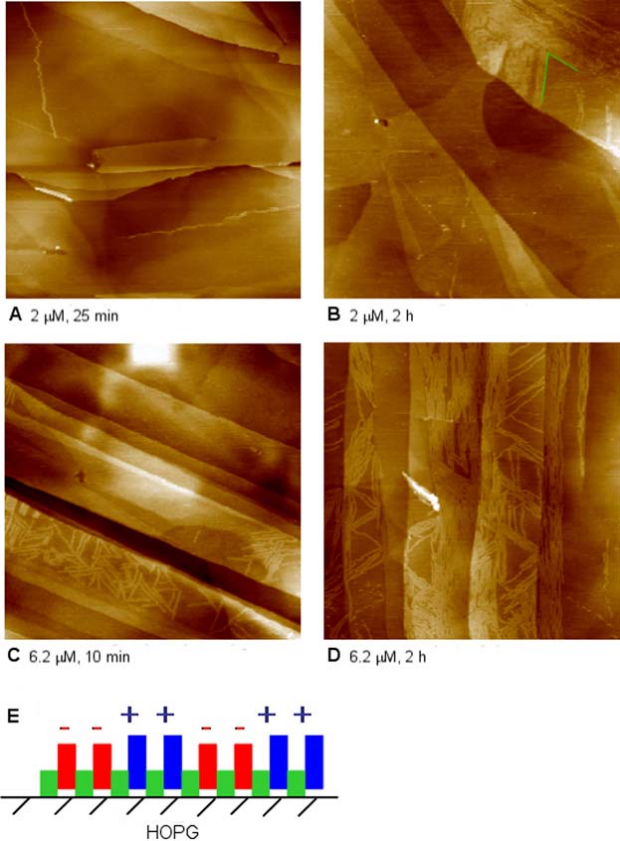
AFM images of EAK 16-II-modified HOPG surface at different concentrations and time periods: (a) 2 µM, 25 min; (b) 2 µM, 2 h; (c) 6.2 µM, 10 min; (d) 6.2 µM, 2 h after different incubation periods. (e) schematic diagram of EAK16-II in a β-strand conformation on HOPG. Each AFM image corresponds to a scan area of 1000 nm×1000 nm.

The AFM images of EAK16-II assemblies formed on HOPG are shown in Figure 4. At a low EAK16-II concentration (2 µM), a few long peptide nanofibers are observed after 25 min incubation time (Figure 4a); a longer incubation time of 2 h results in more nanofibers on the surface, which are either parallel or aligned 60° or 120° to each other (Figure 4b). Such an orientation of the fibers presumably arises due to the hexagonal crystal structure of the HOPG (0001) surface and reflects the importance of the substrate to the peptide nanofiber orientation. When the EAK16-II concentration is increased to 6.2 µM, nanofibers arranged at 60° or 120° to each other are observed within 10 min of incubation (Figure 4c). As the incubation time is increased to 2 h, more nanofibers appear (Figure 4d). Thus, EAK16-II nanofibers can also form on an HOPG surface in a peptide concentration and contact time dependent manner, but with a distribution different from that on mica.

Unlike the case with mica, the affinity of EAK16-II to HOPG is likely due to hydrophobic interactions. As mentioned, the peptide has an amphiphilic structure in which the alanine residues lie on one side of the backbone, while the lysine and glutamic acid residues lie on the other side (Figure 1). When the peptide assembles in a β-strand arrangement [32], it can attach to the surface by a hydrophobic interaction in which the alanine side is oriented toward HOPG and the lysine and glutamic acid groups face the solution, resulting in side-on [35] adsorption (i.e., peptide molecules lie down with their long axes parallel to the substrate surface). Thus, each fiber would be made up of only a single layer of peptides on the surface, unlike the situation with mica where two layers may be stacked on top of one another. (Figure 4e) This reasoning is supported by the observation that the fibers in Figure 4 at both peptide concentrations have a uniform width and height of 6.2±2.0 nm and 0.9±0.2 nm, respectively. A comparison of the height of the patterned nanofibers (0.9±0.2 nm) with that of a single peptide strand (∼0.9 nm) suggests that these fibers consist of single layer β-sheets. If such a structure forms, the EAK16-II-modified HOPG surface should be more hydrophilic than an unmodified surface, as examined further in the next section.

**Figure 5:**
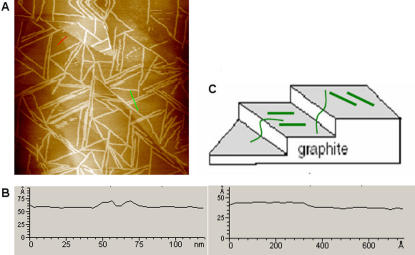
The preferred location of EAK16-II assembled nanofibers on the HOPG surface. (a) A representative AFM image of nanofibers on HOPG surface over a scan area of 800 nm×800 nm; (b) cross-sectional heights of nanofiber on one terrace (green line in a) and a step edge of HOPG (red line in a); (c) schematic diagram of nanofibers on HOPG surface.

Another question to address is whether the self-assembled nanofibers preferably form on the HOPG surface along the edges between adjacent terraces or on the terraces. It is known that the electrodeposition of metal ions occurs preferably along step edges of a surface [36], [37]. In addition, Gettens et al. [35] reported that the protein fibrinogen tends to adsorb on the step edges of HOPG rather than on the terraces. However, the image in Figure 5 indicates that the EAK16-II nanofibers form primarily on the terraces and not at the step edges of HOPG. Figure 5b shows cross-sectional profiles of the HOPG surface along the two line segments indicated in Figure 5a. One line segment (green) crosses two closely-spaced fibers lying on the same terrace of HOPG. This is confirmed by the corresponding cross-section showing that the height of the surface is the same on either side of the two bumps corresponding to the fibers (left plot in Figure 5b). On the other hand, very few of the nanofibers are oriented along the step edges. Similar analysis over other parts of the surface shows that the orientation of the fibers in Figures 4 and 5 is very representative of the overall behavior of the system. A possible explanation for this phenomenon is as follows. When adopting a β-sheet secondary structure, EAK16-II has a unique structure with charged residues on one side and hydrophobic residues on the other side. The strongest interaction of the peptide with a hydrophobic surface would involve contact of the hydrophobic side of the peptide with the terrace of the substrate. Therefore, there is no need for the peptide to selectively align itself along an edge. Another interesting feature of the patterns formed on HOPG is that long EAK16-II nanofibers can actually drape over step edges from one terrace to the next. One advantage of such behavior is that surface defects such as edges and kinks may not significantly inhibit the formation of the EAK16-II nanofibers and their homogeneous distribution over the entire surface.

Previous research has shown that other peptides and proteins can form patterned nanostructures on HOPG surfaces. Examples are elastin-like peptides [38], de novo proteins [39] and amyloid proteins [40]. These patterned nanofibers also tend to align themselves at angles of 60° and 120° to one another. However, the mechanism of protein or peptide association at liquid/substrate interfaces remains unclear.

**Figure 6:**
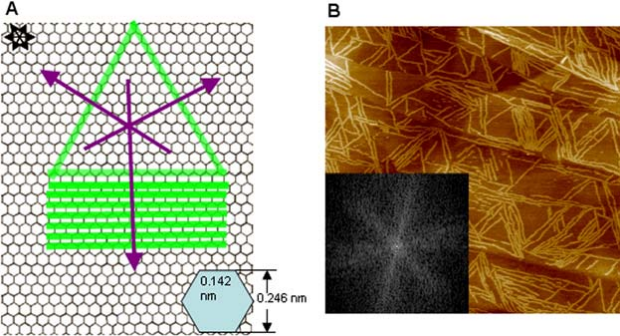
Proposed model of EAK16-II assembly on HOPG. (a) Schematic representation showing the orientation of assembled EAK16-II strands; (b) AFM image of EAK16-II on HOPG surface over a scan area of 1000 nm×1000 nm; the inset shows the 2-dimensional Fourier transform of the AFM image and characteristic 6-fold symmetry.

Based on the analysis of the EAK16-II and HOPG structures, we herein propose a model of EAK16-II assembly into nanofibers on HOPG. The length of an EAK16-II molecule in β-strand is estimated to be ∼6 nm, as discussed in the previous section. This length is comparable to the fiber width observed on the HOPG surface, which is estimated to be ∼6 nm after deconvolution for the AFM tip size effect. In addition, the HOPG surface is made of hexagonal lattices, as shown in Figure 6a. In order to attain the largest hydrophobic contact between EAK16-II and the HOPG surface, it is expected that a peptide molecule would arrange itself to cover as many carbon atoms as possible. From the length of each C-C bond (0.142 nm) in the graphite lattice and the dimension of an EAK16-II β-strand (∼6 nm), one extended strand would cover the length of ∼24 carbon atoms in a row of the graphite lattice (Figure 6a). The peptide can assume the three possible orientations on the HOPG shown as green stripes in order to meet this requirement. Since the peptide strands can line up parallel to one another as they form nanofibers, they can form a network that grows in the directions indicated by the three purple arrows at an angle of 60° or 120° to one another. Such a pattern is very similar to the fiber orientation shown in the AFM image in Figure 6b. The orientation of peptide fibers at 60° or 120° to one another is also indicated by the characteristic 6-fold symmetry of the 2-dimensional Fourier transform of the image (Figure 6b inset).

### Contact angles of EAK16-II modified surfaces

**Figure 7:**
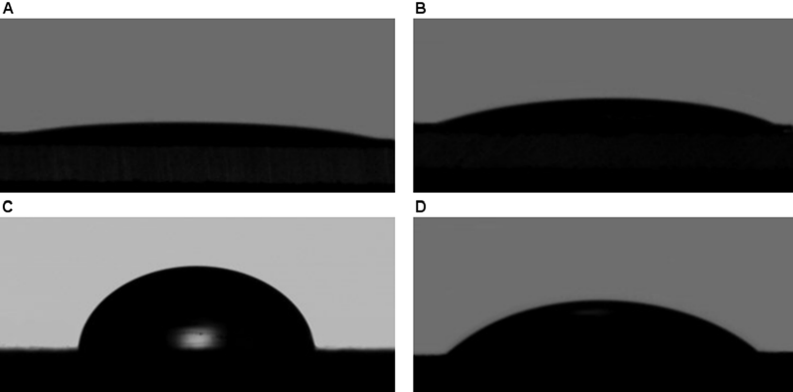
Photographs of water drops on the various surfaces used in the determination of the water contact angles: (a) mica; (b) EAK16-II-modified mica; (c) HOPG; (d) EAK16-II-modified HOPG.

Contact angle measurements were conducted to determine whether the hydrophobicity of mica and HOPG surfaces changes upon modification by EAK16-II. Figure 7 and Table 1 summarize the water contact angle measurements on mica and HOPG surfaces before and after the modification with EAK16-II. The water contact angle on a freshly cleaved mica surface is less than 10° (Figure 7a), indicating that the surface is very hydrophilic and almost completely wettable [41]. Once mica is contacted with EAK16-II and peptide nanofibers form on the mica surface through surface-assisted assembly [34], the water contact angle of the nanofiber coated surface increases to about 20° (Figure 7b and Table 1). This increase could result from the amphiphilic nature of the peptide molecule imparting hydrophobic moieties to the surface upon the modification. However, a water contact angle of 20° indicates that the EAK16-II modified mica surface is still largely hydrophilic. On the other hand, the water contact angle of an EAK16-II-modified HOPG surface decreases to about 39° (Figure 7d). This value is much lower than that obtained on a freshly cleaved HOPG surface, which ranges between 82° and 60° likely due to variations in surface morphology (Figure 7c and Table 1). The significant decrease in contact angle indicates that HOPG becomes more hydrophilic once treated with EAK16-II and is consistent with what would be expected from the proposed single layered β-sheet structure with lysine and glutamic acid oriented toward the aqueous solution (Figure 4e).

**Table 1:** Contact angles measured on mica, HOPG and their EAK16-II-modified surfaces.

surfaces	mica	EAK16-II/mica	HOPG	EAK16-II/HOPG
contact angle	<10°	20.3°±2.9°	71.2°±11.1°	39.4°±4.3°

### Stability of EAK16-II modified surfaces in acidic and alkaline solutions

**Figure 8:**
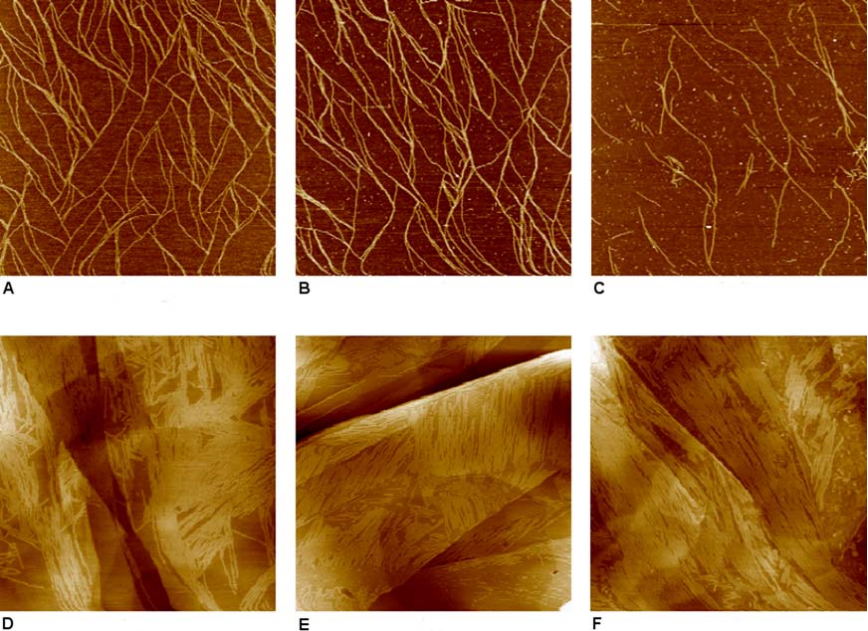
AFM images of EAK16-II modified mica and HOPG surfaces after immersion in pure water, 10 mM HCl and 10 mM NaOH solutions for 10 h. (a) mica, water; (b) mica, 10 mM HCl; (c) mica, 10 mM NaOH; (d) HOPG, water; (e) HOPG, 10 mM HCl; (f) HOPG, 10 mM NaOH. Scan areas are 2000 nm×2000 nm for mica and 1000 nm×1000 nm for HOPG.

To gain more insight concerning the structures of EAK16-II on mica and HOPG surfaces proposed in the previous sections, we investigated the stability of the assembled nanostructures on these two surfaces in acidic and alkaline solutions. Also, in view of the possible practical applications of such modified surfaces, information regarding their stability in different environments would be useful. The stability was monitored in terms of the change in the surface coverage by the EAK16-II. As shown in Figure 8a–c and Table 2, EAK16-II remains on a mica surface as randomly oriented nanofibers in pure water, 10 mM HCl and 10 mM NaOH solutions, although differences in the surface coverage of EAK16-II are observed. After contact of the EAK16-II modified mica surface with the 10 mM HCl solution for 10 h, the surface coverage of nanofibers does not change much (21.4±3.5%) from that obtained prior to contact with the acid (23.7±7.3%). However, the surface coverage decreases significantly to 7.7±3.7% after the modified mica surface has been immersed in a 10 mM NaOH solution for 10 h. These results indicate that an EAK16-II-modified mica surface is more stable in an acidic solution than in an alkaline solution. It has been reported that EAK16-II nanofibers remain stable in the bulk of acidic and alkaline solutions [17]. Thus, the decrease of the amount of nanofibers on the mica surface under alkaline conditions may be due to a weakening of the peptide-surface interaction, resulting from a change of the charge of the EAK16-II residues. Under acidic or neutral conditions, EAK16-II can adsorb on mica through the electrostatic interaction between positively charged lysine and the negatively charged surface. At pH 12 (10 mM NaOH), on the other hand, the lysine residues of EAK16-II become neutral, making the peptide nanofibers negatively charged overall due to the glutamic acid residues. The repulsion between the negatively charged peptide and the negatively charged mica surface would tend to promote the detachment of peptide nanofibers from the mica surface. If this interpretation is correct, it further demonstrates that the electrostatic interaction is a major driving force in the adsorption of EAK16-II onto mica surfaces.

**Table 2:** Peptide coverage on EAK16-II-modified mica and HOPG surfaces after 10 h incubation under acidic and basic environments.

surface coverage %	water	10 mM HCl	10 mM NaOH
mica	23.7±7.3	21.4±3.5	7.7±3.7
HOPG	65.6±7.4	63.9±7.3	65.1±3.8

Note: The peptide coverage on the mica and HOPG surfaces in pure water corresponds to the initial coverage before the surfaces were immersed into an acidic or basic solution. It served as a control for comparison with that of peptide-modified surfaces in acidic and basic environments.

In the case of the EAK16-II-treated HOPG surface, the surface coverage of peptide nanofibers does not change in either the acidic or alkaline environment. As shown in Figure 8d–f and Table 2, the surface coverages of peptide nanofibers are 65.6±7.4%, 63.9±7.3% and 65.1±3.8% after immersion in water, 10 mM HCl and 10 mM NaOH solutions, respectively, for 10 h. These results indicate that the EAK16-II-modified HOPG surface is stable under neutral, acidic and alkaline conditions. This also supports our proposal that the association of the peptide with HOPG surface is mainly through hydrophobic interaction, which should not be strongly affected by the charge state of the peptide. Also, the fact that fibers remain stable on a modified HOPG surface in an alkaline solution, but not on a modified mica surface, provides further support that the loss of stability in the latter case is due to a change in the mica-EAK16-II interaction and not to direct chemical attack on the nanofibers.

Our results have demonstrated that amphiphilic self-assembling peptides are promising materials for surface modification due to the following reasons. First, the modification process occurs spontaneously by adsorption of peptide fibers onto the surface followed by molecular self-assembly to elongate pre-deposited fibers on the surface. This process can be achieved by simply immersing the substrate into an aqueous peptide solution under suitable conditions. Second, it may be possible for other substrates, including gold [42], [43], to be modified by these peptides through various molecular interactions that do not depend on specific thiol-gold chemistry. Third, the peptide sequence can be specially designed and synthesized to incorporate certain amino acids with desired functions.

The advantages of using EAK16-II-modified HOPG surfaces specifically for a number of applications are as follows. First, the hydrophobicity of the modified surface can be significantly reduced to improve its water wettability. The improvement of the water wettability of a given hydrophobic surface by EAK16-II could be utilized to help stabilize hydrophobic compounds in aqueous systems. If successful, this could have significant implications for hydrophobic drug delivery [19], [20]. Second, outward orientation of charged residues (K and E) toward the solution may provide electrostatic interaction and covalent linkage with other analytes and biomolecules so that chemical or enzymatic reactions on the surface can be accelerated. This could be important in electrochemical sensor and biosensor applications as well as the catalysis of biochemical reactions. For example, under certain conditions, covalent bonding can occur between the carboxyl group and deoxyguanosine (dG) residue of DNA [44]. Thus, DNA could be immobilized on a graphite surface with the assistance of EAK16-II assembled nanostructures. Since HOPG is electronically conductive, this modification could allow the fabrication of a new and promising DNA electrochemical sensor. It is also worth noting that the charge state of a peptide or protein can be changed by adjustment of the solution pH. For example, the surface charge of EAK16-II-modified HOPG can be altered to be positive (K) or negative (E) in acidic or basic solutions, respectively, to selectively bind with target molecules. Third, the biocompatibility of the HOPG surface may be improved by EAK16-II, which has been found to support mammalian cell attachment [29].

In conclusion, the ionic-complementary peptide EAK16-II has been shown to assemble on both mica and HOPG surfaces in the form of nanofibers. The interaction between the peptide and mica is mainly through electrostatic attraction, whereas the adsorption of peptides and nanofibers on HOPG occurs through hydrophobic interaction. EAK16-II forms randomly oriented nanofibers with uniform width on the hydrophilic mica surface. In contrast, EAK16-II nanofibers with preferential orientations at angles of 60° or 120° to each other are observed on the hydrophobic HOPG surface. This orientation resembles the crystallographic structure of the graphite surface. After modification with EAK16-II, the contact angle of mica surface increases from below 10° to 20.3±2.9°, but the surface remains hydrophilic. However, the hydrophobicity of the HOPG surface is significantly reduced, as evident from a contact angle change from 71.2±11.1° to 39.4±4.3°. An EAK16-II-modified mica surface is stable in acidic solution, but not in alkaline solution, while the modified HOPG surface is stable in both acidic and alkaline solutions for at least 10 h. This work has shown the potential of using ionic-complementary peptides to modify both hydrophobic and hydrophilic surfaces and may open up the possibility of other surface modifications beyond traditional thiol-gold technology.

## Materials and Methods

### Materials

The ionic-complementary peptide EAK16-II (C_70_H_121_N_21_O_25_, molecular weight 1657 g/mol) has the sequence AEAEAKAKAEAEAKAK (Figure 1) where A corresponds to alanine, E to glutamic acid and K to lysine. At neutral pH, A is neutral, while E and K are negatively and positively charged, respectively. This peptide was purchased from CanPeptide Inc. (Quebec, Canada) with a purity >95% (purified by reverse-phase high-performance liquid chromatography). The N-terminus and C-terminus of the peptide were protected by acetyl and amino groups, respectively.

EAK16-II peptide stock solutions were prepared in pure water (18 MΩ; Millipore Milli-Q system) at concentrations of 12.4 µM and 29 µM. All peptide stock solutions were stored at 4°C before use. Reagent grade sodium hydroxide with purity >99% and hydrochloric acid (36.5–38 wt%) were obtained from BDH Chemicals Ltd. (Toronto, Canada) and Fisher Scientific (Nepean, Canada), respectively.

Grade V-4 muscovite mica (KAl_2_(AlSi_3_)O_10_(OH)_2_) and highly ordered pyrolytic graphite (HOPG, ZYB grade) were obtained from SPI Supplies (West Chester, PA, USA). Mica is negatively charged even at pH 3 [45] and serves as a model hydrophilic surface, whereas HOPG serves as a model hydrophobic surface. Mica and HOPG were first fixed on glass slides using double-sided tape. An adhesive tape was then used to remove the outer layer of mica and HOPG surfaces and expose fresh layers prior to contact with the EAK16-II solutions.

### Preparation of EAK16-II modified surfaces

Appropriate amounts of the 12.4 µM or 29 µM EAK16-II stock solution were diluted to the desired concentrations (1–12 µM) for the experiments to measure the peptide surface coverage on mica and HOPG surfaces. The diluted EAK16-II solutions were added to a liquid cell with a freshly cleaved mica or HOPG surface at the bottom and immobilized for a time period varying from 1 min to 160 min to allow the peptide to adhere and assemble on the surface. The EAK16-II-treated surface was then thoroughly rinsed with pure water three times. To avoid any drying effects on the adherence and assembly of the peptide on the surface, AFM imaging was carried out in pure water.

For contact angle measurements, 30 µl and 300 µl of the 29 µM EAK16-II stock solution were placed on a freshly cleaved mica or HOPG surface (2 cm×2 cm) and allowed to contact each surface for 2 h under a Petri-dish cover to reduce contamination. Each modified surface was then thoroughly rinsed with pure water three times to remove unattached peptides and air-dried prior to measuring the contact angle of a water drop on the surface.

To study the stability of EAK16-II-modified surfaces under different environments, the following procedure was adopted. First, a freshly cleaved mica or HOPG substrate was placed on an AFM sample plate on which an AFM liquid cell was mounted. Then, 500 µl of 4 µM EAK16-II solution was poured into the liquid cell when a mica substrate was used, while 500 µl of a 6 µM EAK16-II solution was contacted with HOPG substrates. These samples were then incubated for ∼10 h to ensure a high degree of EAK16-II surface coverage. During this incubation period, all samples were sealed in an environmental chamber saturated with pure water to avoid evaporation of peptide solution. These EAK16-II treated surfaces were then rinsed with pure water three times to remove unattached peptides. The stability of these EAK16-II-modified surfaces was assessed through AFM images to analyze the changes in peptide surface coverage after exposure to different environments: water, 10 mM HCl and 10 mM NaOH.

### AFM imaging in liquid

The AFM imaging in liquid was performed on a PicoScan^TM^ AFM (Molecular Imaging, Phoenix, AZ) in 500 µl of pure water, a 10 mM HCl or 10 mM NaOH solution (last two solutions were used to assess the stability of EAK16-II-modified surfaces). A scanner with a maximum scan area of 6×6 µm^2^ was used. Silicon nitride cantilevers with a nominal spring constant of 0.58 N/m (DNP-S, Digital Instruments, Santa Barbara, CA) and a typical tip radius of 10 nm were used for tapping mode operation. For the best imaging quality, the tapping frequency was typically set between 16 kHz and 18 kHz and the scan rates controlled between 0.8 and 1 line/s. All experiments were conducted in an environmentally-controlled chamber at room temperature to avoid evaporation of the solution. All AFM images were obtained at a resolution of 256×256 pixels.

### Quantitative analysis of AFM images

The width and height of EAK16-II nanofibers on mica and HOPG surfaces were determined using the cross-sectional analysis tool of the PicoScan software. The peptide fiber widths reported herein were obtained using the deconvolution method reported by Fung et al [30]. The surface coverage of EAK16-II on mica and HOPG was analyzed with Image J software (http://rsb.info.nih.gov/ij/). For each sample, at least five images (6 µm×6 µm) at different locations were analyzed to obtain the average surface coverage. The AFM tip deconvolution was not performed in estimating the surface coverage of the peptide assemblies. Therefore, the surface coverage values reported in this paper are apparent values and higher than the true values. This is sufficient for this study since our objective was to show the trends on the effect of incubation time and peptide concentration on the coverage of EAK16-II nanofibers on mica and HOPG surfaces. Another purpose of the surface coverage measurements is to compare the stability of EAK16-II-modified surfaces in various environments.

### Contact angle measurement

Contact angle measurements were made to determine changes in the hydrophobicity of the surfaces upon their modification by EAK16-II. A sessile drop of water (30 µl) was manually deposited on the unmodified and EAK16-II-modified surfaces placed inside a sealed, water-saturated chamber (to prevent evaporation). A sequence of images of the water drop was then recorded over a period of 5 minutes in 5-second intervals. The recorded images were analyzed by the Axisymmetric Drop Shape Analysis-Profile (ADSA-P) program [46] to yield a water contact angle. All the experiments were controlled by a refrigerated bath/circulator digital controller (NESLAB Instrument, Inc., Georgetown, Ontario) to ensure that the temperature remained at 20±0.1°C during the measurements. Static water contact angles for each surface are reported herein and represent the average measured values obtained from three different samples.
